# Identification of DmTTLL5 as a Major Tubulin Glutamylase in the *Drosophila* Nervous System

**DOI:** 10.1038/s41598-017-16586-w

**Published:** 2017-11-24

**Authors:** Isabelle Devambez, Juliette van Dijk, Salim Benlefki, Sophie Layalle, Yves Grau, Krzysztof Rogowski, Marie-Laure Parmentier, Laurent Soustelle

**Affiliations:** 1 IGF, CNRS, INSERM, Univ Montpellier, Montpellier, France; 2 CRBM, CNRS, Univ Montpellier, Montpellier, France; 3 IGH, CNRS, Univ Montpellier, Montpellier, France; 4 Present Address: The Institute for Neurosciences of Montpellier, INSERM, Saint Eloi Hospital, Montpellier, France

## Abstract

Microtubules (MTs) play crucial roles during neuronal life. They are formed by heterodimers of alpha and beta-tubulins, which are subjected to several post-translational modifications (PTMs). Amongst them, glutamylation consists in the reversible addition of a variable number of glutamate residues to the C-terminal tails of tubulins. Glutamylation is the most abundant MT PTM in the mammalian adult brain, suggesting that it plays an important role in the nervous system (NS). Here, we show that the previously uncharacterized CG31108 gene encodes an alpha-tubulin glutamylase acting in the *Drosophila* NS. We show that this glutamylase, which we named DmTTLL5, initiates MT glutamylation specifically on alpha-tubulin, which are the only glutamylated tubulin in the *Drosophila* brain. In *DmTTLL5* mutants, MT glutamylation was not detected in the NS, allowing for determining its potential function. *DmTTLL5* mutants are viable and we did not find any defect in vesicular axonal transport, synapse morphology and larval locomotion. Moreover, *DmTTLL5* mutant flies display normal negative geotaxis behavior and their lifespan is not altered. Thus, our work identifies DmTTLL5 as the major enzyme responsible for initiating neuronal MT glutamylation specifically on alpha-tubulin and we show that the absence of MT glutamylation is not detrimental for *Drosophila* NS function.

## Introduction

The formation and the correct functioning of a complex tissue such as the nervous system require multiple microtubule-mediated processes. Indeed, microtubules (MTs), which constitute one of the major cytoskeletal components of neurons, play important roles during the establishment and the maintenance of neuronal polarity^1^, the regulation of neuronal morphology^2^ and the formation of synaptic connections^3,4^. In addition, MTs act as highways for transport of proteins, mRNAs and organelles to cell compartments distant from the neuronal cell body^5^. Also, the importance of MT cytoskeleton in neuronal development and physiology is further supported by the fact that MT defects are responsible for a wide range of human neurodevelopmental disorders and neurodegenerative diseases^6–8^. MTs carry out their multiple cellular functions by interacting with many microtubule-associated proteins (MAPs). Amongst them, members of the structural MAP family stabilize MTs and counteract the effects of MT-severing enzymes such as Spastin or Katanin^9^. In addition, molecular motors of the Kinesin and Dynein families mediate, respectively, anterograde and retrograde transport of cargoes^5^.

MTs are composed of heterodimers of alpha and beta-tubulins whose carboxy-terminal regions project outward from the MT surface making them accessible for the interaction with MAPs and motor proteins^10^. Such interactions may be modulated by variations in the C-terminal tails of tubulin isotypes, which generate functional MT heterogeneity^11^. Indeed, the C-terminal tails of tubulins are the only region showing variability between the different members of alpha or beta-tubulins isotypes^12^. MT heterogeneity is further generated by diverse post-translational modifications (PTMs), which are particularly abundant at the carboxy-terminal regions of tubulins^13^. Thus, functional properties of MTs may be regulated by structural diversity at the tubulin C-terminal tails.

Amongst all PTMs occurring on tubulins, glutamylation is a PTM in which a chain of variable length composed of glutamate residues is attached to the carboxy-terminal region of both alpha and beta-tubulins. The first glutamate is added through a γ-linked isopeptide bond to a glutamate residue of tubulin protein and additional glutamates are then added to the growing chain via standard peptide bonds^14^. MT glutamylation, which was first reported in 1990^15^, is evolutionarily conserved from protists to mammals^15–17^. It is estimated to affect about 40 to 50% of alpha-tubulin present in the mouse brain^15^. For comparison, another PTM of alpha-tubulin, the acetylation at Lys40, represents less than 5% of total tubulin in the mouse brain^18^. Thus, MT glutamylation is a PTM highly enriched in neurons.

Enzymes catalyzing MT glutamylation belong to the Tubulin Tyrosine Ligase Like (TTLL) family^19^. In mammals, nine genes encoding glutamylases have been identified. When over-expressed in mammalian cells, some of them preferentially catalyze glutamylation on alpha-tubulin while the others prefer beta-tubulin^19–21^. In addition, some TTLLs initiate tubulin glutamylation by adding the first glutamate, while others elongate the chain by adding glutamate residues to the first one^19–21^. In these experiments, three TTLLs (TTLL1, 2 and 9) were inactive, strongly suggesting that they act in a complex as it was shown for TTLL1^19,21^. The mammalian TTLL family also includes three other members that encode enzymes, called glycylases, initiating or elongating glycine chains on tubulins^22^.

The *Drosophila melanogaster* genome contains eleven genes belonging to the TTLL family^19^. While two of them (DmTTLL3A and DmTTLL3B) are involved in glycylation of MTs in the *Drosophila* testis^22^, the role of the remaining TTLL-encoding genes is unknown. A previous study has shown that only alpha-tubulin is glutamylated in the *Drosophila* nervous system^23^. However the identification of *Drosophila* glutamylases and whether neuronal MT glutamylation plays important role remain to be determined. The aim of our study was to identify the enzyme initiating MT glutamylation in order to investigate *in vivo* the role of MT glutamylation in the *Drosophila* nervous system.

Here, we show that the previously uncharacterized *CG31108* gene is orthologous to the mammalian *TTLL5* gene and encodes an enzyme initiating MT glutamylation on alpha-tubulin in the *Drosophila* nervous system. In *DmTTLL5* mutant brains, glutamylation on alpha-tubulin is absent and rescue experiments confirm that DmTTLL5 initiates glutamate chains specifically on alpha-tubulin. Thus, DmTTLL5 is a major enzyme responsible for neuronal MT glutamylation. Flies lacking neuronal MT glutamylation are viable and show no defects in vesicular axonal transport, synapse morphology nor locomotion. In addition, *DmTTLL5* mutant flies have similar lifespan as compared to wild-type flies, reflecting no major dysfunction of the nervous system. Thus, our data suggest that neuronal MT glutamylation, which is initiated on alpha-tubulin by DmTTLL5, is not essential for the correct functioning of the *Drosophila* nervous system.

## Results

### CG31108 is the *Drosophila* ortholog of mammalian TTLL5

A previous study has shown that MTs were glutamylated specifically on alpha-tubulin in the *Drosophila* brain^23^. Thus, to study the role of neuronal MT glutamylation, we wanted to identify the enzyme involved in the initiation of glutamate chains, hypothesizing that fly mutant for this enzyme will lack MT glutamylation.

In the mouse brain, glutamylation on alpha-tubulin is carried out by TTLL1, which has two orthologous genes in the *Drosophila melanogaster* genome (*CG8918* and *CG32238*)^19^. However, Flybase data (http://flybase.bio.indiana.edu/) indicate that these two genes are not expressed in the nervous system, making them not relevant candidates. The only other murine TTLL initiating glutamylation on alpha-tubulin is TTLL5^21^. BLAST searches at FlyBase identified the uncharacterized *CG31108* gene as the *Drosophila* ortholog of mouse *TTLL5* ^19^. As *CG31108* is expressed in the nervous system (Flybase data), we focused our attention on this gene. Based on Flybase, *CG31108*, which we named *DmTTLL5*, produces two transcripts encoding identical protein.

In order to determine whether the DmTTLL5 protein contains the functional motifs found in all TTLLs, we aligned its sequence with its murine ortholog (Supplementary Figure 1). These two proteins share 30% and 29% of identical and similar residues, respectively. As expected, the highest conservation between these two proteins is found in the regions corresponding to the core TTL and the extended TTL domains (Supplementary Figure 1). The core TTL domain contains essential ATPase site found in all TTLL proteins while the extended TTL domain is present in TTLL enzymes acting as glutamylases but absent in the ones acting as glycylases^21^. Interestingly, the DmTTLL5 protein also carries a cationic MT binding domain (c-MTBD) that is required for MT binding and glutamylation activity^24^. This c-MTBD is found in all TTLL glutamylases with autonomous activity^24^, suggesting that DmTTLL5 does not function as part of a complex like it is thought for mammalian TTLL1, 2 and 9^19,21,25^.

Taken together, DmTTLL5, encoded by the *CG31108* gene, contains all the characteristic domains found in the glutamylase members of the TTLL family.

### Glutamylation on alpha-tubulin is absent in the brain of DmTTLL5 mutants

**Figure 1:**
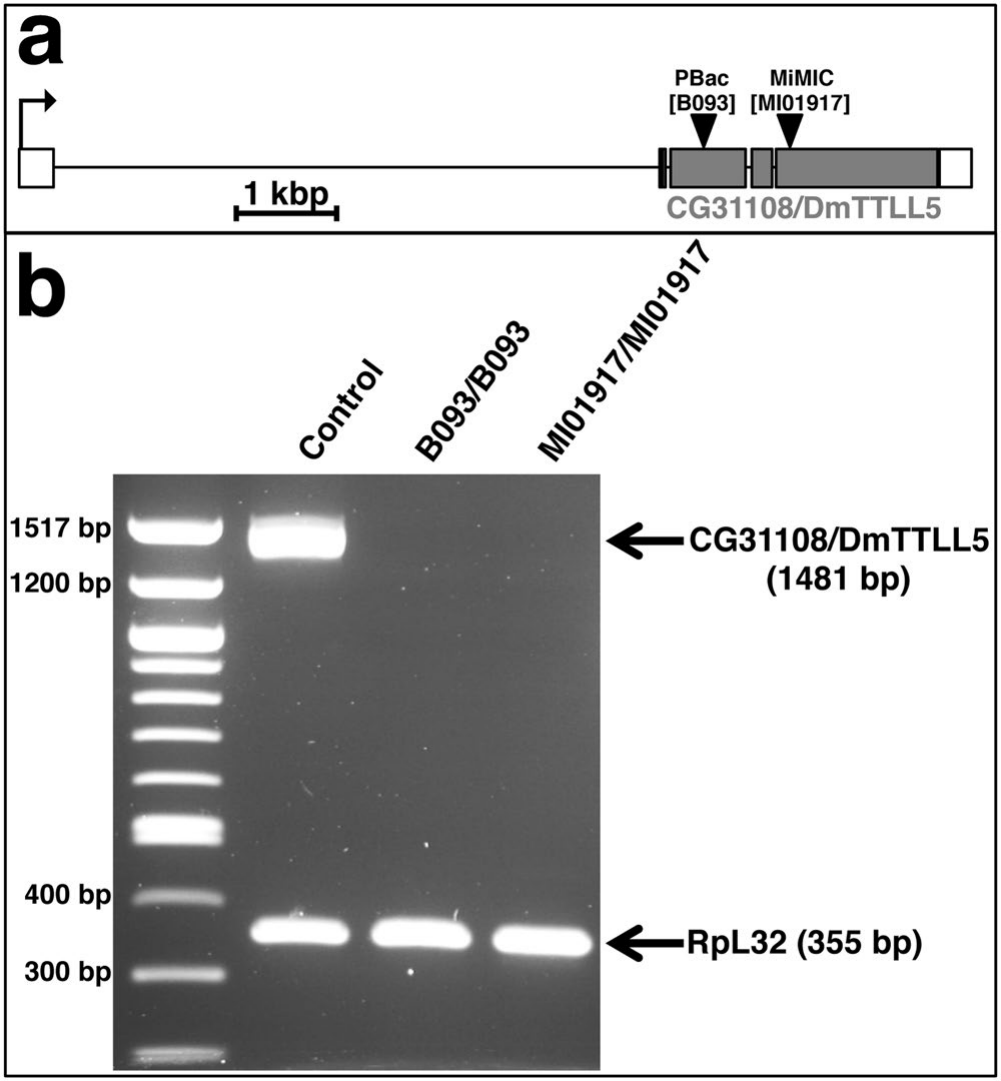
Characterization of two *CG31108/DmTTLL5* mutant lines. (**a**) Schematic representation of the *CG31108/DmTTLL5* gene based on Flybase data for CG31108-RA transcript. Another transcript, called CG31108-RB, has a shorter 3′ UTR and codes for the same protein (not shown). Non-coding and coding exons are shown in white and grey, respectively. Black arrow indicates the orientation of transcription. The insertion sites of PBac[B093] and MiMIC[MI0917] transposons are indicated by black arrowheads. Note that both transposons are inserted into the coding sequence of *CG31108/DmTTLL5*. (**b**) RT-PCR analysis of total RNA prepared from adult heads of control and flies homozygous for B093 or MI01917 insertions. *CG31108/DmTTLL5* is expressed in the heads of control flies but not in the ones of *CG31108/DmTTLL5* ^*B093/B093*^ and *CG31108/DmTTLL5* ^*MI10917/MI01917*^ mutants. Note that the expression level of *Ribosomal protein L32* (RpL32) mRNA is unchanged between control and *CG31108/DmTTLL5* mutants.

To study the function of *DmTTLL5*, we obtained two distinct *Drosophila* strains carrying transposon (called B093 and MI01917) inserted within its open reading frame (Fig. 1a), which could therefore affect *DmTTLL5* expression and function. To confirm that DmTTLL5 was expressed in *Drosophila* brains and to address if its expression was indeed affected by the presence of B093 and MI01917 insertions, we performed RT-PCR experiments from *Drosophila* heads, as these two insertions were homozygous viable. As shown in Fig. 1b, *DmTTLL5* mRNA is present in adult head of control flies. In contrast, no *DmTTLL5* transcript was detected in both homozygous mutant lines (B093/B093 and MI01917/MI01917 in Fig. 1b), strongly suggesting that these two mutants are *DmTTLL5* null mutants.

**Figure 2:**
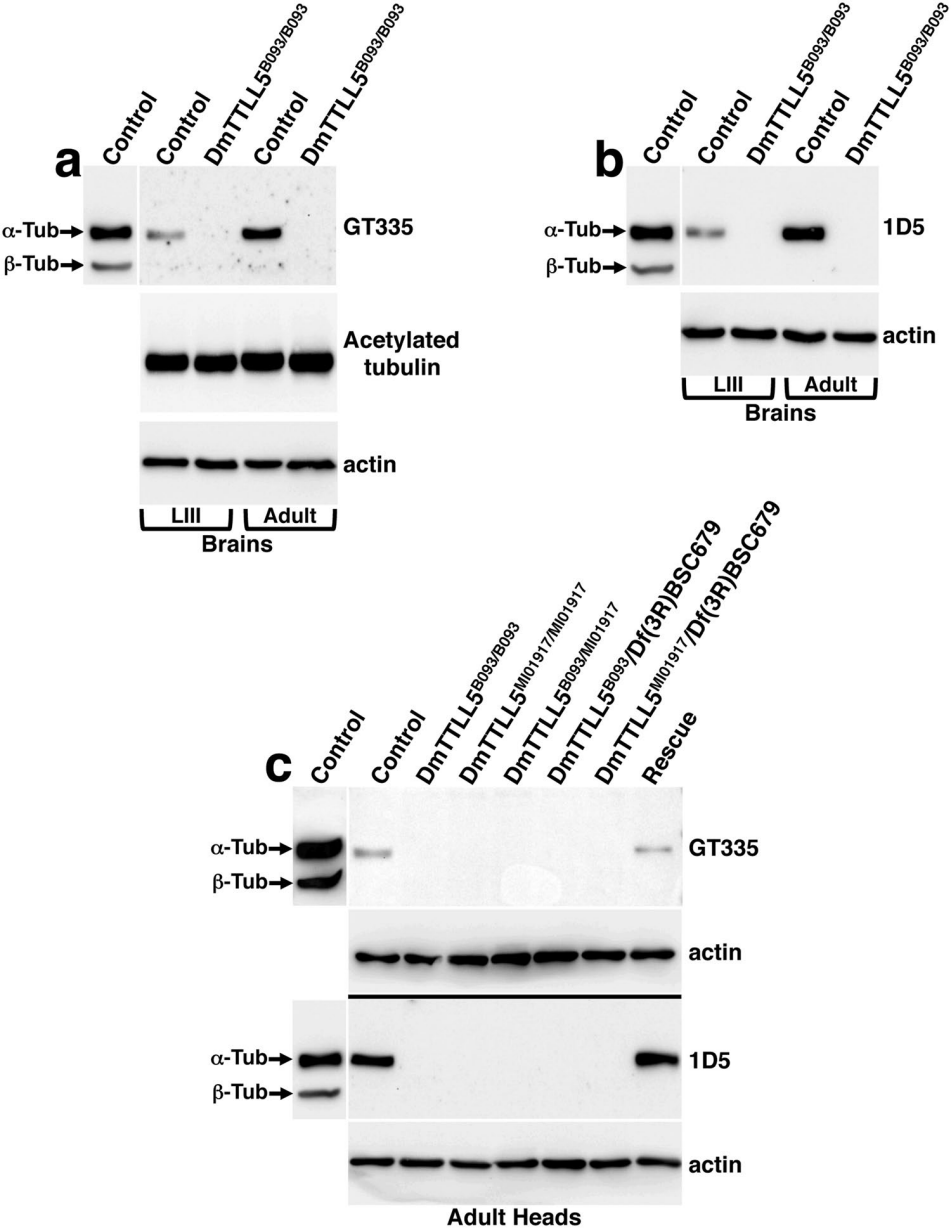
DmTTLL5 is required for alpha-tubulin glutamylation in the *Drosophila* nervous system. (**a**–**c**) Representative immunoblots on protein extracts from larval (LIII in a,b) or adult dissected brains (Adult in **a,b**) and *Drosophila* heads (**c**). For all panels, anti-actin was used as loading control; DM1A and E7 antibodies were used to detect alpha and beta-tubulin, respectively. Western blots were cropped in this figure; full blots are shown in Supplementary Figure 2–4. (**a**,**b**) GT335 (**a**) and 1D5 (**b**) antibodies were used to analyze MT glutamylation in larval and adult *Drosophila* brains of control and *DmTTLL5* ^B093/B093^ mutant. In control, MTs are glutamylated on alpha-tubulin but not on beta-tubulin while no signal is visible in *DmTTLL5* ^B093/B093^ mutant. Note that the levels of acetylated alpha-tubulin in the brains of control and *DmTTLL5* ^B093/B093^ mutant are similar **(a)**. **(c)** By using GT335 (up panel) or 1D5 (bottom panel) antibodies, MT glutamylation is detected on alpha-tubulin in the heads of control but not in the different *DmTTLL5* mutant backgrounds (*DmTTLL5* ^B093^ homozygous, *DmTTLL5* ^MI01917^ homozygous, *DmTTLL5* ^B093/MI01917^ transheterozygous, as well as *DmTTLL5* ^B093^ and *DmTTLL5* ^MI01917^ transheterozygous over the Df(3 R)BSC679 deficiency). Df(3 R)BSC679 is a deficiency deleting thirteen genes including *DmTTLL5*. In rescue condition (elav-Gal4/+; UAS-DmTTLL5-EYFP/+; DmTTLL5^B093/B093^ indicated by Rescue), MTs are glutamylated on alpha-tubulin but not on beta-tubulin.

Before analyzing the state of MT glutamylation in *DmTTLL5* mutants, we wanted to confirm that glutamylation only occurs on alpha-tubulin in the *Drosophila* nervous system. For this purpose, we first performed immunoblot analyses on protein extracts from larval and adult brains by using GT335 antibody, which specifically recognizes the branch points of glutamate side chains and detects all glutamylated forms of target proteins, independently of the length of glutamate chains^26^. As shown in Fig. 2a, MT glutamylation was detected on alpha-tubulin but not on beta-tubulin in larval as well as adult brains. To strengthen this data, we then used the 1D5 antibody (also known as ID5), which recognizes glutamate chains of three or more residues on both alpha and beta-tubulins^27^. By using 1D5 antibody, MT glutamylation in larval and adult brains was also observed only on alpha-tubulin (Fig. 2b). Thus, in the *Drosophila* central nervous system (CNS), MTs are glutamylated on alpha-tubulins only and at least part of them contains glutamate chains of more than three residues.

To analyze whether MT glutamylation was affected by the loss of *DmTTLL5* function, we first performed immunoblot experiments on brain extracts from *DmTTLL5* ^B093^ homozygous mutants. As shown in Fig. 2(a,b), MT glutamylation was not detected in the larval and adult brains of such mutants by using either GT335 or 1D5 antibodies. We also examined the level of acetylated alpha-tubulin and did not find any difference between control and *DmTTLL5* mutants (Fig. 2a), indicating that loss of *DmTTLL5* function has no effect on this PTM. Then, to confirm that loss of MT glutamylation was effectively due to the loss of *DmTTLL5* function, we analyzed several *DmTTLL5* mutant genetic backgrounds by using protein extracts from adult heads. As was observed from dissected brains, alpha-tubulin was glutamylated in control but not in *DmTTLL5* ^B093/B093^ adult heads (Fig. 2c), indicating that MT glutamylation in adult heads is dependent on *DmTTLL5* function. By using GT335 or 1D5 antibodies, we also did not detect MT glutamylation in the heads of flies homozygous for the MI01917 insertion as well as *DmTTLL5* ^B093^/*DmTTLL5* ^MI01917^ transheterozygous mutant flies (Fig. 2c). Similar results were obtained with flies carrying one copy of B093 or MI01917 insertion over a deficiency deleting *DmTTLL5* plus twelve other genes (Fig. 2c). Thus, in all *DmTTLL5* mutant conditions, we did not detect glutamylation on alpha-tubulin.

To definitively conclude that the lack of MT glutamylation observed in *DmTTLL5* mutant flies was indeed due to the loss of *DmTTLL5* function, we performed rescue experiments. For this purpose, we used the pan-neuronal elav-Gal4 driver line to express *DmTTLL5* in all neurons of *DmTTLL5* ^B093/B093^ mutant flies. By using GT335 and 1D5 antibodies, we showed that MT glutamylation on alpha-tubulin was restored in rescued flies and that no signal was observed on beta-tubulin, as in control flies (Fig. 2c).

Altogether, these results show that, in the nervous system, DmTTLL5 is a major glutamylase initiating glutamylation specifically on alpha-tubulin.

### Microtubules are glutamylated in larval segmental nerves and presynaptic motorneuron endings

**Figure 3:**
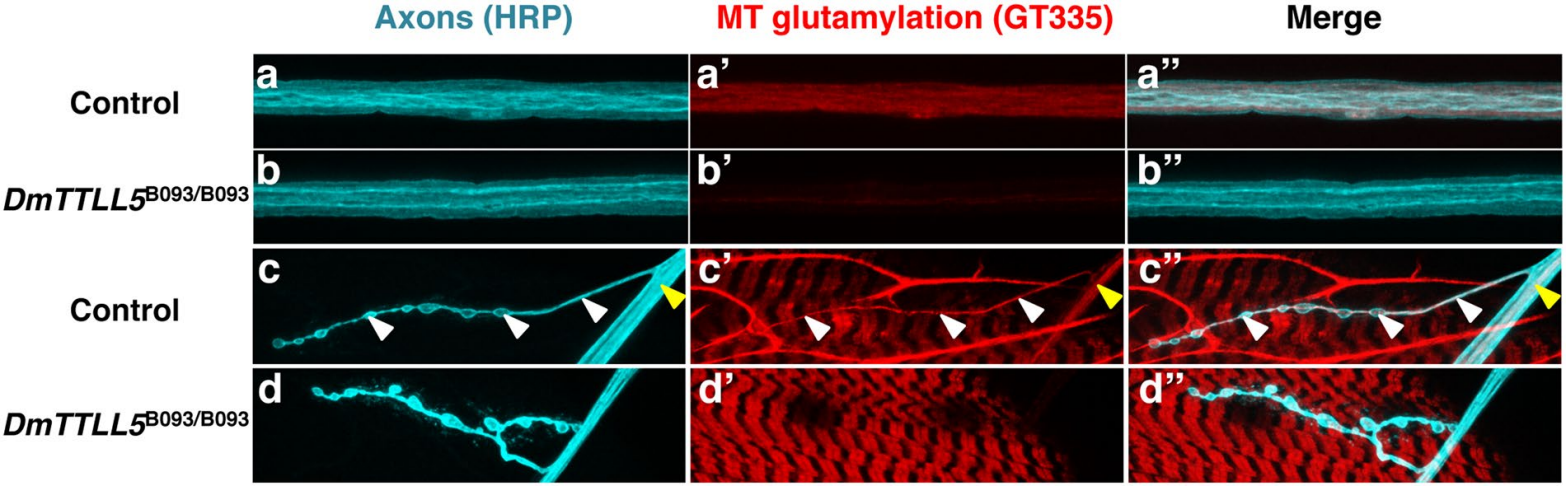
Glutamylation of microtubules in axons and presynaptic motorneuronal endings of *Drosophila* larvae. (**a**–**d”**) anti-HRP (cyan in **a**,**b**,**c**,**d**) and GT335 (red in **a’**,**b’**,**c’**,**d’**) co-labeling of nerves (**a**,**b**”) and neuromuscular junctions (**c**,**d”**) from *Drosophila* third instar larvae. The merged panels are shown on the right (**a**”,**b**”,**c**”,**d**”). (**a-a”**) In control, glutamylated MTs (**a’**) are present throughout the axons, which are labeled with the neural marker anti-HRP (**a**). (**b-b”**) In the axons of *DmTTLL5* ^*B093/B093*^ mutant larvae, MTs are not glutamylated as indicated by the absence of GT335 immunoreactivity (see **b’**). (**c,d”**) At the neuromuscular junctions of control (**c**-**c**”), glutamylated MTs are present at the presynaptic endings of motorneurons (white arrowheads in **c**-**c**”) but not in *DmTTLL5* ^*B093/B093*^ mutants (**d’**,**d**”). The yellow arrowhead in (**c**-**c**”) indicates the glutamylated MTs in motorneuron nerve. Note that a GT335 signal is also present in muscles but this labeling is independent of *DmTTLL5*. The HRP-negative fibers in (**c’**) are autofluorescent tracheal cells that are present in control preparation.

By using GT335 antibody, we performed immunostaining on larval and adult brains but the labeling was diffuse and did not reveal any particular labeled structures (data not shown). However, a clear signal was detected in the axons of larval segmental nerves (Fig. 3a-a”), which contain the axons of motorneurons and peripheral neurons. In *DmTTLL5* mutant, a dramatic decrease of MT glutamylation was observed (Fig. 3b-b”), indicating that DmTTLL5 is involved in MT glutamylation in larval segmental nerves. GT335 signal was also found at the larval neuromuscular junction (NMJ) in the presynaptic motorneuron endings (Fig. 3c-c”) and this staining is specific as it was absent in *DmTTLL5* mutant (Fig. 3d-d”). Thus, the glutamylation of MTs is present in nerves of larval motorneurons as well as in their presynaptic endings that contact muscles.

### Larval neuromuscular junction organization and morphology are not affected in DmTTLL5 mutant

**Figure 4:**
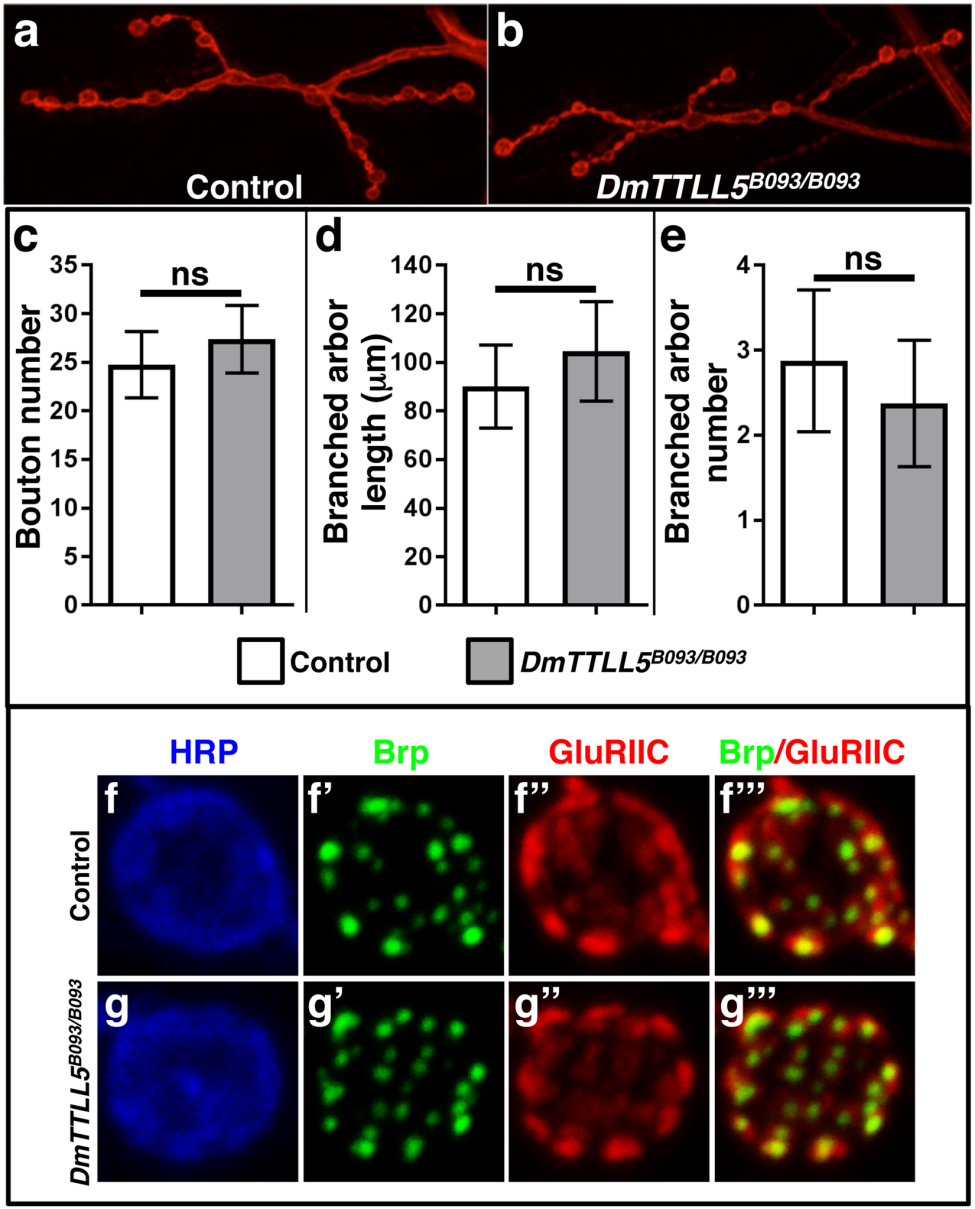
Absence of alpha-tubulin glutamylation has no influence on larval neuromuscular junction morphology. (**a**,**b**) Third instar larval neuromuscular junctions of control (**a**) and *DmTTLL5* ^*B093/B093*^ mutant (**b**) labeled with anti-HRP antibody (in red). (**c–e**) Quantification of synaptic bouton number (**c**), branched arbor length (**d**) and number (**e**). No statistical differences were observed between control (white bars) and *DmTTLL5* ^B093/B093^ mutant (grey bars) larvae (p > 0.05, Unpaired two-tailed parametric t test, n between 8 and 11). (**f,g”’**) High magnification showing a bouton labeled with anti-HRP (in blue), anti-Bruchpilot (Brp, in green) and anti-GluRIIC (in red) antibodies. Merge of anti-Brp and anti-GluRIIC labeling are shown in (**f**”’,**g**”’). In control (**f**-**f**”’) and in *DmTTLL5* ^*B093/B093*^ mutant larvae (**g**-**g**”’), all active zones have both Brp (pre-synaptic) and GluRIIC (post-synaptic) punctae juxtaposed to each other.

Larval NMJ development and morphology are affected by a variety of proteins that alter MT dynamics^28,29^ and several studies have shown that the level of glutamylation may influence MT dynamics^30–32^. Thus, the presence of MT glutamylation in motorneurons incited us to analyze whether the morphology of larval NMJs was affected in *DmTTLL5* mutants. As observed with anti-HRP staining, which detects neuronal membranes^33^, wild-type larval NMJs are organized into branched arbors composed of chains of synaptic boutons (Fig. 4a). In absence of MT glutamylation, the NMJs were indistinguishable from the control (Fig. 4b). We quantified the number of boutons, the length and the number of branch arbors and did not find any difference compared to the control (Fig. 4c–e).

At larval NMJs, stable MTs are necessary to promote and maintain the active zones^34,35^, which are the sites of neurotransmitter release apposed to glutamate receptor clusters. So, we asked whether MT glutamylation might play a role in synaptic organization. Each bouton houses several active zones identifiable by the presence of the Bruchpilot proteins, which are juxtaposed to the post-synaptic glutamate receptors including GluRIIC (Fig. 4f-f””). In absence of DmTTLL5, the organization of the active zones was not altered as each presynaptic Brp staining was found at the vicinity of post-synaptic GluRIIC staining (Fig. 4g-g”’). Thus, the loss of MT glutamylation does not seem to alter the morphology or the synaptic architecture of larval NMJs.

### Vesicular axonal transport is not affected by lack of microtubule glutamylation

**Figure 5:**
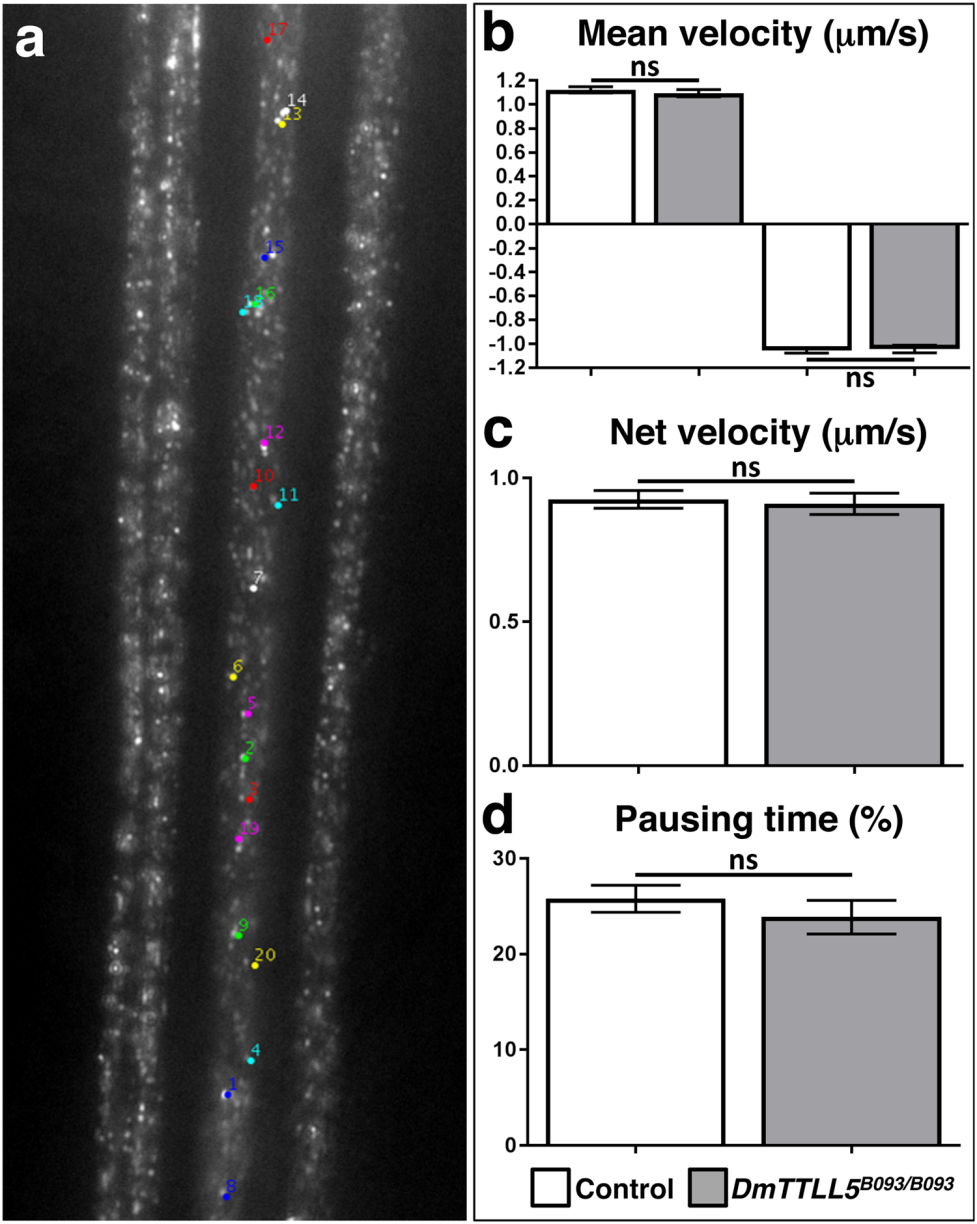
Vesicular axonal transport is not impaired in absence of glutamylation on alpha-tubulin. (**a**) First image of a representative movie of vesicular movement along segmental nerves of third instar *Drosophila* larva. Twenty vesicles are randomly selected for analyses (colored dots). (**b**–**d**) Kinetic parameters of vesicular movement in segmental nerves of control (OK6-Gal4;UAS-NPYGFP, white bars) and *DmTTLL5* mutant (OK6-Gal4;UAS-NPYGFP::DmTTLL5^B093/B093^, grey bars). The mean instant velocity of vesicles moving anterogradely (positive values in **b**) or retrogradely (negative values in **b**) as well as the mean net distance run by a vesicle during 1 second (net velocity in **c**) are shown. The percentage of time during which vesicles are not moving is indicated in (**d**, pausing time). ns indicates no significant statistical difference between the two genotypes (p > 0.05, Unpaired two-tailed non parametric t test-Mann-Whitney test; between 155 and 277 vesicles analyzed).

A recent *in vitro* study using chimeric tubulins has revealed that the level of glutamylation may induce subtle defects on molecular motor activities^36^. Thus, we analyzed the axonal transport of vesicles by using a non-invasive tracking of vesicles in motorneurons nerves. For this purpose, a neuropeptide Y-GFP (NPYGFP) construct was expressed in all motorneurons by using the OK6-Gal4 driver. We then tracked the motion of individual fluorescent vesicles, through the cuticle of anesthetized living larvae allowing us to quantify different parameters of vesicle kinetics (Fig. 5a). In OK6-Gal4;UAS-NPYGFP control larvae, the mean instant velocity of vesicles moving anterogradely or retrogradely were 1.12 ± 0.03 μm and 1.05 ± 0.02 μm per second, respectively (Fig. 5b). The total net distance run in 1 sec (net velocity) was 0.93 ± 0.03 μm (Fig. 5c) while the pausing time of vesicles was 25.8 ± 1.4% (Fig. 5d). In *DmTTLL5* mutant larvae (OK6-Gal4;UAS-NPYGFP::DmTTLL5^B093/B093^), the mean instant velocity of anterograde and retrograde transports as well as the net velocity of vesicles were not statistically different compared to the control (Fig. 5b,c). In addition, the pausing time of vesicles in *DmTTLL5* mutant was slightly reduced but not statistically different compared to the control (Fig. 5d). Thus, it seems that the vesicular axonal transport in larval motorneurons was not affected in absence of glutamylation.

### Locomotion behavior and adult life span are not impaired in DmTTLL5 mutant

**Figure 6:**
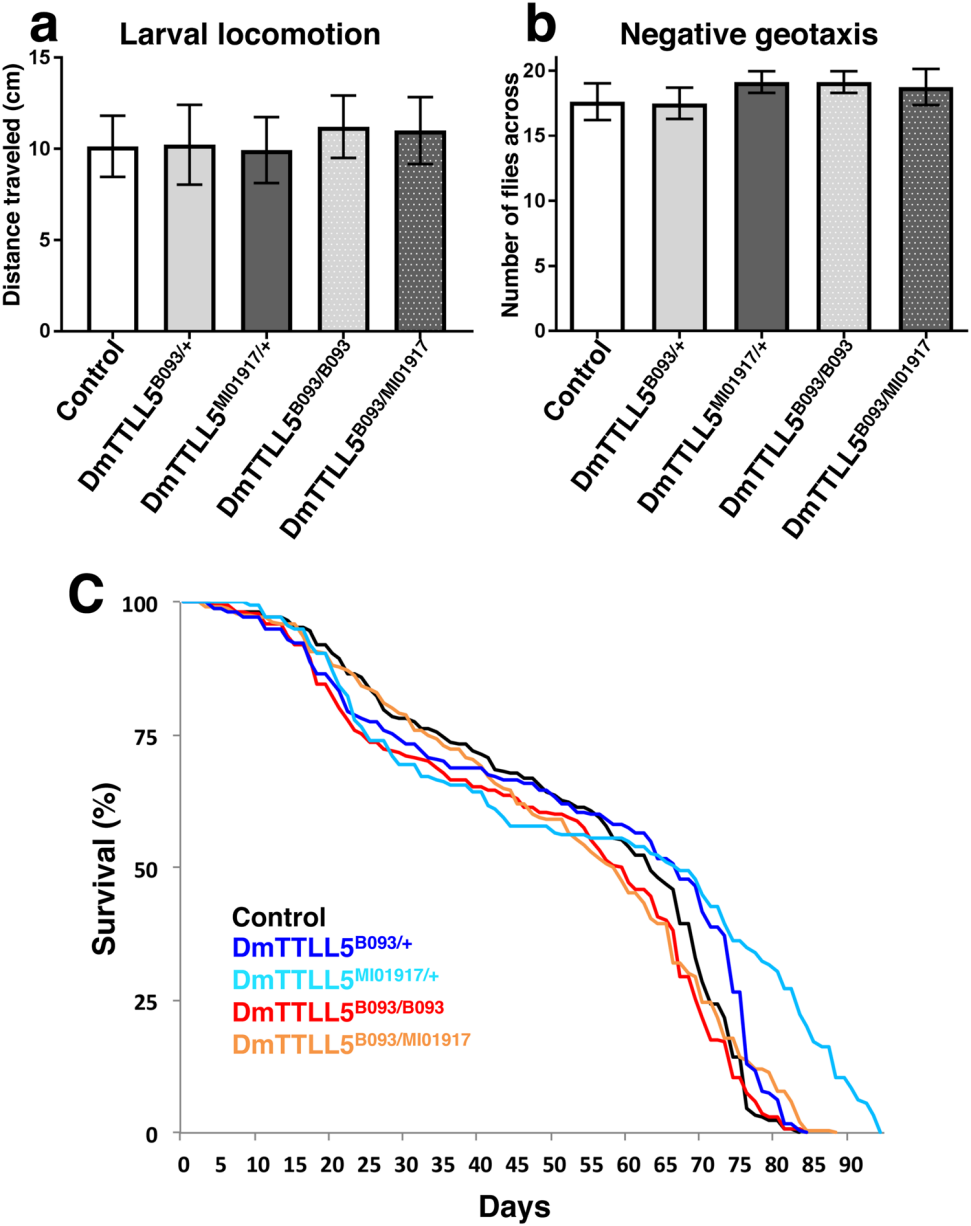
Locomotion as well as life span are not altered in *DmTTLL5* mutants. (**a**) The distances traveled by wild-type (control in white) and heterozygous control (*DmTTLL5* ^*B093*^/+ in light grey and *DmTTLL5* ^*MI01917*^/+ in dark grey) larvae are not statistically different compared to the ones of *DmTTLL5* mutants (*DmTTLL5* ^*B093/B093*^ in dotted light grey and *DmTTLL5* ^*B093/MI0917*^ in dotted dark grey). p > 0.05, Kruskal-Wallis non parametric multiple comparison test, n = 15 for each genotype. (**b**) Negative geotaxis behavior of controls (wild-type control in white, *DmTTLL5* ^*B093/*+^ in light grey, *DmTTLL5* ^*MI01917/*+^ in dark grey) as well as *DmTTLL5* mutants (*DmTTLL5* ^*B093/B093*^ in dotted light grey, *DmTTLL5* ^*B093/MI01917*^ in dotted dark grey) adult flies. No significant statistical differences were found between all genotypes (p > 0.05, One-way ANOVA Tukey’s multiple comparisons test, n = 8 for each genotype). (**C**) Analysis of adult lifespan for controls (wild-type control, *DmTTLL5* ^*B093/*+^ in dark blue, *DmTTLL5* ^*MI01917/*+^ in light blue) as well as *DmTTLL5* mutant (*DmTTLL5* ^*B093/B093*^ in red, *DmTTLL5* ^*B093/MI01917*^ in orange) flies is shown. Survival curves of controls and *DmTTLL5* mutant flies are comparable.

One major function of the nervous system is to elicit behavior such as locomotion, and subtle neuronal defects may be revealed by abnormal behavioral output. To address whether lack of MT glutamylation may have an effect on larval locomotion, we analyzed the traveled distance by larvae placed in a Petri dish during 2 min. In wild-type control larvae, the mean traveled distance was 10.15 ± 1.67 cm (Fig. 6a) and we did not observe difference for *DmTTLL5* ^*B093*^/+ and *DmTTLL5* ^*MI01917*^/+ heterozygous control larvae (Fig. 6a, 10.23 ± 2.19 and 9.94 ± 1.81 cm, respectively). Compared to these controls, *DmTTLL5* ^*B093/B093*^ mutant larvae did not show statistically different results (Fig. 6a, 11.22 ± 1.71 cm). To strengthen this observation, we tested another *DmTTLL5* mutant background, *DmTTLL5* ^*B093/MI01917*^ trans-heterozygous, and did not find statistically differences compared to controls (Fig. 6a, 11 ± 1.84 cm). Thus, lack of MT glutamylation does not alter larval locomotion.

Then, we assessed adult locomotion and climbing ability by using a negative geotaxis assay. This assay, which is elicited by mechanical stimulation of the flies, measures an innate escape response in which flies ascend the wall of a container after being tapped to its bottom. Compared to wild-type and heterozygous controls, *DmTTLL5* ^B093^ homozygous and *DmTTLL5* ^B093/MI01917^ trans-heterozygous mutant flies did not show impairment of negative geotaxis behavior (Fig. 6b), indicating that their locomotion and climbing ability are not affected in absence of MT glutamylation.

Generally, major dysfunction of the nervous system is associated with reduced lifespan and most if not all neurodegenerative *Drosophila* mutants show decreased viability^37^. In order to determine whether *DmTTLL5* mutant flies may display neuronal degeneration, we compared the lifespan of *DmTTLL5* ^B093^ homozygous as well as *DmTTLL5* ^B093/MI01917^ trans-heterozygous flies with control flies. As shown in Fig. 6c, the absence of tubulin glutamylation has no impact on fly survival, suggesting that neuronal integrity is not affected.

Altogether, these data indicate that the loss of *DmTTLL5* function does not alter locomotion or lifespan, strongly suggesting that the absence of tubulin glutamylation is not detrimental for nervous system function and maintenance.

## Discussion

MT glutamylation is a PTM highly enriched in the nervous system from *Drosophila* to mammals^38^, suggesting that this PTM plays important role(s) in neuronal development, function or homeostasis. In this study, we identified DmTTLL5, encoded by the *CG31108* gene, as a major MT glutamylase in the *Drosophila* CNS. Our data show that DmTTLL5 initiates MT glutamylation specifically on alpha-tubulin, the only glutamylated tubulin in the *Drosophila* CNS, as we could not detect glutamylation on beta-tubulin by using two different antibodies. Our data show that *DmTTLL5* mutant flies, which lack detectable level of glutamylated tubulin in their nervous system, do not seem to have major neuronal dysfunction as synapse morphology, vesicular axonal transport, locomotion and lifespan are not altered.

In mammalian nervous system, TTLL1 and TTLL7 are considered as the major glutamylases^19–21^. TTLL1 (also called PGs3) is a member of a glutamylase complex including, at least four other components (PGs1, 2, 4 and 5)^19,39,40^. In this complex, only TTLL1 acts as the catalytic subunit with glutamylase activity showing a preference for alpha-tubulin^19^. In the brains of TTLL1 knock-out (KO) mice, a strong reduction of glutamylated MTs was observed^41^. However, TTLL1 KO mice have normal embryonic and post-natal development^42,43^, do not show any discernable lesions in the CNS and perform normally neurobehavioral tests^43^. This suggests that TTLL1 function is dispensable in mammalian neurons^19^. Alternatively, it is possible that residual MT glutamylation in TTLL1 KO mice is sufficient for correct neuronal function. ROSA22 mice are mutants for PGs1, a member of the TTLL1-including glutamylase complex that has no glutamylase activity but acts to localize the glutamylase complex^40,44^. It was shown that the levels of polyglutamylated as well as tyrosinated alpha-tubulin were highly decreased in the brain of ROSA22 mice^45^. However, glutamylation was not totally absent as the levels of monoglutamylated alpha-tubulin were unchanged^45^. In ROSA22 mice, the KIF1A kinesin, but no other kinesins (KIF3A, KIF5A) was abnormally distributed in neurites leading to a modest reduction of the synaptic vesicle density at synaptic terminals and a decrease of continuous synaptic transmission^45^. While these data suggested that such defects might be due to the decrease of alpha-tubulin polyglutamylation, the authors did not exclude that the mislocalization of kinesin may be due to the reduction of tyrosinated tubulin or to a mechanism independent of the status of tubulin PTMs^45^. In this way, it is important to keep in mind that CLIP170 is drastically reduced in neurite extensions when tyrosinated tubulin is decreased^46^. CLIP170, which preferentially binds to tyrosinated tubulin, plays important role in axonal transport^47^. Thus, all the defects observed in ROSA22 mice may be due to the mislocalization of CLIP170. It was also observed that, contrary to wild-type mice, ROSA22 male mice did not exhibit intermale aggressive behavior^44^. However, whether this phenotype is due to nervous system defects or other tissues that express PGs1 remains to be determined^44^. To summarize, it is still unclear whether the neural phenotypes observed in ROSA22 mice are really due to a decrease of MT glutamylation.

TTLL7, which is a glutamylase specific to beta-tubulin, is also highly expressed in the adult murine brain^20^. A previous study has analyzed the function of TTLL7 in neuron-like PC12 cells. While in naïve neuronal PC12 cells, TTLL7 is not expressed and beta-tubulin is not glutamylated, Nerve Growth Factor (NGF)-induced differentiation of PC12 cells leads to TTLL7 expression and beta-tubulin glutamylation^20^. Interestingly, knockdown of TTLL7 during NGF-induced PC12 cell differentiation blocks beta-tubulin glutamylation and reduces neurite growth, suggesting a causal relationship between the activity of TTLL7 and neurite formation^20^. However, TTLL7 knockdown in primary culture of mouse superior cervical ganglion (SCG) neurons, which express TTLL7, had no effect on neurite growth^20^. This discrepancy may be explained by an unusual role of TTLL7 in PC12 cells or by compensatory mechanisms in SCG neurons. Further, *in vivo* studies on the role of TTLL7, especially the generation of TTLL7 KO mice, are necessary to better understand the role of beta-tubulin glutamylation in the nervous system. Thus, altogether with our data, it seems that in mammalian as well as in *Drosophila* neurons, a reduction of the glutamylated MT levels is not detrimental for neuronal life and function. However, we cannot exclude that some neuronal aspects or complex behaviors, not explored in this study may be affected by lack of MT glutamylation.

By using quantitative immunoblotting on *Drosophila* brains with two different antibodies (GT335 and 1D5), we did not observe glutamylation on beta-tubulin isotypes. Thus, from our data and those of Bobinnec and collaborators^23^, we may conclude that MT glutamylation occurs predominately on alpha-tubulin isotypes in the *Drosophila* CNS. Of course, we cannot completely rule out that some beta-tubulin isotypes are glutamylated at extremely low undetectable level. The lack of beta-tubulin glutamylation in the *Drosophila* CNS is somewhat surprising as both alpha and beta-tubulins are glutamylated in the mammalian brain. It is thought that all glutamate residues present in the C-terminal tails of tubulins may be potentially glutamylated but the experimentally verified modified glutamate (E) of mammalian beta-tubulin is always found within the following protein sequences: YED, GEF and AEE^48^. The *Drosophila* genome contains four genes encoding beta-tubulin. While the YED and AEE motifs are not present in *Drosophila* beta-tubulins, the GEF motif is present and conserved at the same position in only one beta-tubulin: beta-tub85D. Interestingly, it was shown that beta-tub85D, which is specifically expressed in testis^49^ (and Flybase data) is glutamylated^50^. From these observations, it appears that the modified glutamate residue is distinguished by their neighboring amino acids generating consensus sites of glutamylation. Accordingly, the Glu445 residue that is glutamylated in murine alpha1a and alpha1b-tubulins is the last glutamate in the GEGE motif, which is conserved in alpha-tubulins expressed in *Drosophila* heads (alpha-Tub84B and alpha-Tub84D).

As we observed for DmTTLL5, murine TTLL5 initiates glutamylation preferentially on alpha-tubulin^21^. However, mammalian TTLL5 is an unusual member of the TTLL family as the murine and human proteins contain an additional C-terminal domain of approximately 400 residues that is absent in all other TTLLs^21,51^. This C-terminal domain, also called receptor interaction domain, is involved in many transcriptional cofactor activities related to glucocorticoid receptor-modulated transcription, explaining that MmTTLL5 is also called STAMP (SRC-1 and TIF2 associated modulatory protein)^51^. It is very unlikely that DmTTLL5 possesses such transcriptional cofactor activity, as the protein does not contain this additional C-terminal domain (Supplementary Figure 1). It was recently found that mutations in human *TTLL5* are associated with retinal dystrophy^52,53^, and two recent studies have identified the gene encoding retinitis pigmentosa GTPase regulator (RPGR) as a specific physiological substrate of mammalian TTLL5^54,55^. RPGR is one of the genes commonly mutated in retinitis pigmentosa, as RPGR mutations account for around 20% of all retinitis pigmentosa cases^56^. RPGR is expressed in a complex pattern with at least ten alternative transcripts^56^. One of them, called RPGR^ORF15^, is strongly expressed in the retina^57^. The RPGR^ORF15^ protein is characterized by a unique C-terminal exon called ORF15 that contains a glutamic acid-glycine (Glu-Gly)-rich acidic domain^56^, which could potentially be substrate for glutamylation, and a basic C-terminal domain. A previous study has demonstrated that MmTTLL5, but no other MmTTLLs, binds to the basic domain of RPGR^ORF15^ protein and then glutamylates several sites in the (Glu-Gly)-rich acidic domain^55^. In addition, it was shown that aged *TTLL5* KO mice develop retinal phenotype similarly to *Rpgr* KO mice, strongly suggesting that the absence of RPGR glutamylation abolished its function. In *Drosophila*, it is very unlikely that such a pathway exists for several reasons. While orthologous sequences of RPGR exist in invertebrates, the C-terminal exon ORF15 found in vertebrates does not exist in invertebrates including *Drosophila* ^58^. Thus, the invertebrate RPGRs lack the domain that is normally glutamylated by MmTTLL5. Most importantly, MT glutamylation is not affected in the retina of *MmTTLL5* KO mice, indicating that the retinal dystrophy phenotype is specifically due to the loss of RPGR glutamylation^55^. Finally, MmTTLL5 is also expressed in the nervous system but, to our knowledge, no neural phenotypes were described to date. Thus, future studies need to be done to reveal the function of MmTTLL5 in the mammalian nervous system.

The aim of our study was to use *Drosophila* to investigate *in vivo* the role of MT glutamylation in the nervous system. However, no obvious neuronal defects have been detected in *DmTTLL5* mutant flies, suggesting that MT glutamylation seems not to be essential for neuronal or axonal function. However, recent *in vitro* studies have found that MT glutamylation may influence several MT-linked proteins^31,36,59^. For example, it was shown that the length of glutamate chains modulates the activity of the MT-severing enzyme Spastin^31^. Indeed, Spastin activity gradually increases with the number of glutamate added per tubulin and then decreases above a threshold. Interestingly, similar observations were described for the affinity of the Tau protein to MTs, even if the threshold was not the same^60^. Overall, it seems that the variable length of glutamate chains branched onto tubulins is the key signal modulating the activity of proteins acting on MTs. In this way, we may speculate that MTs are kind of neutral in absence of glutamylation, explaining why no major defects were found in *DmTTLL5* mutants. Another way to explain that lack of neuronal MT glutamylation has no dramatic effect is that the functions of MAPs and molecular motors are also modulated by various PTMs^61,62^. So, it is possible that in absence of MT glutamylation, neurons compensate by acting on such PTMs to adjust the function of MT associated proteins, which could not be the case *in vitro*. In the future, *Drosophila* will be an excellent model to decipher *in vivo* whether the function of such MT associated proteins could be modulated by glutamylation.

## Materials and Methods

### Drosophila stocks

The wild-type control strain was wCS, (*w* ^*1118*^ flies outcrossed with CantonS for 10 generations, kindly provided by J.M. Dura, IGH, Montpellier, France). The elav-Gal4^c155^ (B#458) driver, the B093 (B#16140) and MI01917 (B#32800) insertions and the Df(3 R)BSC679 deficiency (B#26531) stocks were obtained from Bloomington *Drosophila* stock center. OK6-Gal4 and UAS-NPY stocks were kindly provided by C. O’Kane and I. Robinson, respectively (University of Cambridge, UK). For axonal transport analyses, a recombinant stock was created between UAS-NPYGFP and B093 insertions. The UAS-DmTTLL5-EYFP fly strain was made by site-specific insertion into the attP40 landing site (The Bestgene Company). DmTTLL5 sequence was amplified by PCR (forward primer: 5′ CGCGGAGAATTCACCATGCCTTCTTCATTGTGTGAAGCTCTC 3′; reverse primer: 5′ CGCCGCAGATCTTAGAAATACCTTCTCCTTGTCGAAGTTC 3′) from a home-made *Drosophila* testis cDNA library and inserted into pEYFP-N1 vector via EcoRI/BamHI. The DmTTLL5-EYFP cDNA was then cut out of this vector with EcoRI/NotI and inserted into pAttB-UAS vector. To exclude any genetic background effect, *DmTTLL5* ^B093^ and *DmTTLL5* ^MI01917^ flies were outcrossed to wCS flies for five generations before to perform behavioral experiments.

### RT-PCR experiments

Total RNAs from 50 adult heads were extracted in 500 μl of Trizol (Invitrogen) following manufacturer’s instructions. Reverse transcription was performed with M-MuLV Reverse Transcripase (Biolabs) and random nonanucleotide primers (RP9 from Biolabs) following manufacturer’s instructions. A multiplex PCR reaction was performed with the following primers: *DmTTLL5* 5′-GTTTCGCCGAAGTCGTTATG-3′ and 5′-CCTGCAGGCCGAAATAATAG-3′, housekeeping gene *RpL32* 5′-CTAAGCTGTCGCACAAATGG-3′ and 5′-CCACGTTACAAGAACTCTCA-3′. Multiplex PCR was done in a MasterCycler (Eppendorf) as follows: 10 min at 95 °C followed by 35 cycles: 30 sec at 95 °C, 45 sec at 65 °C, 90 sec at 68 °C and a final step of 5 min at 68 °C. Volume reaction was 50 μl and 15 μl were used for analyze on 1% agarose gel. DNA ladder was 100 bp DNA ladder from BioLabs (#N3231S).

### Immunoblots

Protein extracts were prepared from heads or dissected brains. Cell homogenization was done in RIPA buffer (150 mM sodium chloride, 1% NP-40, 0.5% sodium deoxycholate, 0.1% sodium dodecyl sulphate, 50 mM Tris, pH 8.0, supplemented with protease inhibitor cocktail from Roche Life Science). After 2 hrs of lysis at room temperature, Laemmli buffer was added before to boil the samples during 5 min.

Samples were analyzed by SDS-PAGE. To separate alpha and beta-tubulins, we used a protocol described by Banerjee and collaborators^63^. Separated proteins were electrophoretically transferred onto nitrocellulose membrane (Hybond C-Extra, Amersham Biosciences) prior to blotting. Primary and secondary antibodies were incubated in 5% milk in PTX (PBS, 0.1% TritonX100) and washes were done with PTX. Immunodetection was done with Clarity Western ECL kit (BIO-RAD). Chemiluminescence detection was acquired with ChemiDoc Touch Imaging System (BIO-RAD). The following primary antibodies were used: mouse GT335 (1/200, AdipoGen), mouse 1D5 (1/500, Synaptic System), mouse anti-acetylated-tubulin (1/2000, Sigma), mouse DM1A (1/4000, anti-alpha tubulin from Sigma), mouse E7 (1/1000, anti-beta tubulin from DSHB), mouse anti-actin (1/2000, ThermoScientific). Secondary antibody used was HRP-linked goat anti-mouse (1/10000, Jackson ImmunoResearch).

### Immunocytochemistry

Third instar larvae were dissected in 1 mM EDTA containing PBS and then fixed 20 min at room temperature in 4% paraformaldehyde. After several washes with PTX (PBS, 0.3% TritonX100), samples were incubated in (0.2% BSA in PTX) containing primary or secondary antibodies. Washes were done in PTX. When using anti-GluRIIC antibody (kindly provided by A. DiAntonio, Washington University Medical School, United States), the same protocol was used except for tissue fixation that was done in Bouin’s solution (Sigma) during 15 min. The following primary antibodies were used: mouse GT335 (1/1000), goat anti-HRP Cy3 or Cy5 (1/1000, Jackson ImmunoResearch), mouse anti-Brp (1/200, nc82 from DSHB), rabbit anti-GluRIIC (1/3000). Secondary antibodies (1/800, Jackson ImmunoResearch) included donkey anti-mouse Cy3, donkey anti-mouse Alexa Fluor 488, donkey anti-rabbit Cy3. Samples were mounted in 80% glycerol. Images were acquired by using a confocal microscope (Leica SPE or Zeiss LSM780).

### Vesicular axonal transport

The tracking of vesicles in segmental nerves of third instar larvae was performed as described in^64^. Briefly, larvae were anaesthetized with ether during 2 min and mounted, with their ventral face up, in polymerizing 1% agarose between a slide and a cover slip. The tracking of vesicle motion was done on segmental nerves in A2-A3 segments under a 63X oil objective of an upright wide field fluorescent microscope. For each larva, one nerve was chosen and a movie of 100 frames taken every 280 ms was recorded. Then, each movie was analyzed by selecting 20 vesicles on the first fixed frame (i.e. before to know if vesicles were moving or not) that were then tracked with the imageJ plugin ‘manual vesicle tracking’ developed by F. Cordelières (Institut Curie, Orsay, France). As a control, larvae expressing NPY-GFP in motor neurons (OK6-Gal4;UAS-NPYGFP) were used and compared to the *DmTTLL5* mutant genotype (OK6-Gal4;UAS-NPYGFP::DmTTLL5^B093/B093^). The percentage of pausing corresponds to the percentage of time during which vesicular instant velocity is inferior to 0.4 μm/s. The mean distance (anterograde and retrograde) corresponds to the total distance traveled by a vesicle divided by the time during this vesicle was analyzed (expressed in μm/sec).

### Larval locomotion assay

Third instar larvae were individually placed at the center of a petri dish filled with grape juice agar, classically used for *Drosophila* egg collection. Larval locomotion was video-recorded during 2 min with a camera (Sony HDR-CX240) positioned above assay petri dish and movies were analyzed with the imageJ plugin ‘manual vesicle tracking’ developed by F. Cordelières (Institut Curie, Orsay, France). For each genotype, 15 larvae were analyzed.

### Negative geotaxis assay

One-week-old flies were anaesthetized under CO2 and 20 flies (10 males and 10 females) of each genotype were placed in 25 ml BD falcon pipets cut at one end and filled with cotton. Flies were allowed to recover for 2 hours before testing. Then, each pipet was assayed by gentle tapping to engage the negative geotaxis assay and each assay was repeated twice with 2 min intervals. After 45 sec, flies having reached the top of the column (above 22 cm) were counted. For each genotype, four independent groups of flies were tested.

### Survival analyses

For lifespan analysis, 60 once-mated female flies of each genotype were distributed in 6 tubes containing food, leading to 10 females per tube. Then, the number of dead flies was counted every 2–3 days when transferred on fresh food. All lifespan analyses were performed at 25 °C. Results are expressed as the percentage of survivors from four cohorts (three for *DmTTLL5* ^MI01917^/+ flies) analyzed at different time.

### Statistical analysis

All data are shown as means ± S.D. D’Agostino-Pearson omnibus normality test was used to test for normal distribution (with alpha value = 0.05). Data with normal distribution were analyzed with Unpaired two-tailed parametric t test. For axonal transport analyses, Unpaired two-tailed non parametric t test (Mann-Whitney test) was used. Kruskal-Wallis non parametric multiple comparison test and one-way ANOVA Tukey’s multiple comparison test were used for larval locomotion and negative geotaxis analysis, respectively. All statistical analyses were performed using GraphPad Prism 7.03 Software (San Diego, CA, USA).

### Data availability

All data generated or analyzed during this study are included in this article (and its Supplementary Information files). All reagents are available upon request.

### Ethics Statement

Invertebrate animals, i.e. *Drosophila*, were used that is exempted from ethics approval.

## Electronic supplementary material

Suplementary information
