# Supplementary material for: Identification of DmTTLL5 as a Major Tubulin Glutamylase in the Drosophila Nervous System
Source: Sci Rep. 2017 Nov 24;7:16254. doi: 10.1038/s41598-017-16586-w (PMC5701211; doi:10.1038/s41598-017-16586-w)
Supplement: Supplementary file 1 — Suplementary information [file 41598_2017_16586_MOESM1_ESM.pdf]

## SUPPLEMENTARY FIGURES

# IDENTIFICATION OF DmTTLL5 AS A MAJOR TUBULIN GLUTAMYLASE IN THE DROSOPHILA NERVOUS SYSTEM.

Isabelle DEVAMBEZ[1], Juliette van DIJK[2], Salim BENLEFKI[1,3], Sophie LAYALLE[1], Yves GRAU[1], Krzysztof ROGOWSKI[4], Marie-Laure PARMENTIER[1], Laurent SOUSTELLE[1,*]

[1] IGF, CNRS, INSERM, Univ Montpellier, Montpellier, France
[2] CRBM, CNRS, Univ Montpellier, Montpellier, France
[3] Present address: The Institute for Neurosciences of Montpellier, INSERM, Saint Eloi Hospital, Montpellier, France
[4] IGH, CNRS, Univ Montpellier, Montpellier, France.

* Correspondence should be addressed to L.S.
(Laurent.Soustelle@igf.cnrs.fr)

# Supplementary Figure 1

```
MmTTLL5         ------------------------------------------------------------       0
DmTTLL5         MPSSLCEALTNSSISFDYQKEDDWITSGKLGSREAVLVFRTNILNPKIRKTLSEKSSAQV       60

MmTTLL5         ------------------------------------------------------------       0
DmTTLL5         LTDTKDNEEKPVQSPQKLAEEEPTSSEPSSKEALSGSTQQKIFLLKKPQQCDKSDKLTFS       120

MmTTLL5         --MPVVMAR-DLEETASSSEDEDLANQEDHPCIM-----------------WTGGCRRI-       39
DmTTLL5         SPFKKILNRYSSSDTSISSEGEEPANSSENKSRVLKTSQNAINIKLATDLSNESGYESIA       180
                  :  :: *  . .:*:  *** *: **..:. . :                .*  . *

MmTTLL5         PVLVFHAEAI----------LTKDNNIRVIGERYHLSYKIVRTDSRLVRSILTAHGFHEV       89
DmTTLL5         PAKTISNTEVIEEDQEETSEDDAECGINMPASKLKITYKFHQTETKLLRKLFNVHGLTEV       240
                *. .:    :              :  *.: ..: :::**: :*:::*:*.::..**: **

MmTTLL5         HPSSTDYNLMWTGSHLKPFLLRTLSEAQKVNHFPRSYELTRKDRLYKNIIRMQHTHGFKA       149
DmTTLL5         QGENNNFNLLWTGVHMKLDIVRNLAPYQRVNHFPRSYEMTRKDRLYKNIERMQHLRGMKH       300
                : ...::**:*** *:*  ::*.*:  *:*********:********** ****  :*:*

MmTTLL5         FHILPQTFLLPAEYAEFCNSYSKDRGPWIVKPVASSRGRGVYLINNPNQISLEENILVSR       209
DmTTLL5         FDIVPQTFVLPIESRDLVVAHNKHRGPWIVKPAASSRGRGIFIVNSPDQIPQDEQAVVSK       360
                *.*:****:** *  ::  ::.*.********.*******::::*.*:**  :*: :**:

MmTTLL5         YINNPLLIDDFKFDVRLYVLVTSYDPLVIYLYEEGLARFATVRYDQGSKNIRNQFMHLTN       269
DmTTLL5         YIVDPLCIDGHKCDLRVYVLVTSFDPLIIYLYEEGIVRLATVKYDRHADNLWNPCMHLCN       420
                **  :** ** .* *:*:******:***:*******:.*:***:**: :.*: *  *** *

MmTTLL5         YSVNKKSGDYVSCDDPEVEDYGNKWSMSAMLRYLKQEGKDTTALMAHVEDLIIKTIISAE       329
DmTTLL5         YSINKYHSDYIRSSDAQDEDVGHKWTLSALLRHLKLQSCDTRQLMLNIEDLIIKAVLACA       480
                **:**  .**: ..* : ** *.**::**:**:** :. **  ** .:******::::.

MmTTLL5         LAIATACKTFVPHRSSCFELYGFDVLIDNTLKPWLLEVNLSPSLACDAPLDLKIKASMIS       389
DmTTLL5         QSIISACRMFVPNGNNCFELYGFDILIDNALKPWLLEINLSPSMGVDSPLDTKVKSCLMA       540
                 :* :**: ***. ..********:****:*******:*****:. *:*** *:*:.::

MmTTLL5         DMFTVVGFVCQDPAQRTSNRSIYPSFESSRRNPFQKPQRTRPLSASDAEMKNLVASAREK       449
DmTTLL5         DLLTCVGIPAYSPEMKSHYDQKWSRFRSSSCQRIATF----P--STSQ--------KTKK       586
                *::* **: . .*  ::   . :  *.**  : : .     *  ::.         :*

MmTTLL5         VPGKLGGSVLGLSMEEIKVLRRVKEENDRRGGFIRIFPTSETWEIYGSYLEHKTSMNYML       509
DmTTLL5         SKKKGAAINLNLTGEEQRILRNARLQYSRRGGFVRIFPTDDSMQRYGNFLDSANGIPIST       646
                   *  ..  * *: ** ::**..: : .*****:*****.:: : **.:*:   ..:

MmTTLL5         ATRLFQDRGNPRRSLLTGRARVSTEGAPELKVESMNSKAKLHAALYERKLLSLEVRKRRR       569
DmTTLL5         PNVQSQTF-----------------QTPI---IQHNYNQMLHQNLYSKD-----GRQKQ-       680
                 .   *                  :*     . * :  **   **.:.      *:::

MmTTLL5         RSGRLRAMRPKYPVIAQPAEMNIKTETESEEEEEVGLDNDDEEQEASQEESAGSLGENQA       629
DmTTLL5         ----------------------------------------DDNNEAD----------RIW       690
                                                        *:::**.          .

MmTTLL5         KYTPSLTVIVENSPRDNAMKVAEWTNKGEPCCKIEAQEPESKFNLMQILQDNGNLSKVQA       689
DmTTLL5         QYERALET-------DSEI--PFVKKPTVEKCEVEGRR--LRKIMLKKISNGSELTPFQA       739
                :*  :* .       *. :     .:     *::*.:.  :  ::: :.: .:*: .**

MmTTLL5         RLAFSAYLQHVQIRLTKDSGGQTLSPSWAAKEDEQMELVVRFLKRASSNLQHSLR-MVLP       748
DmTTLL5         RQTFSMYLESVLRRLTEDPKD------------NHEKIILKFLNKFGGSVKPPVMFRNMQ       787
                * :** **: *  ***:*               :: :::::**:: ...:: :   :

MmTTLL5         SRRLALLERRRILAHQLGDFIGVYNKETEQMAEKKSKKKLEEEEEDGVNAESFQEFIRQA       808
DmTTLL5         TIKVASKARSAMVAKLLGDFLENYKRDTEAYVDSFDHF-------GMIPSSAYNQFLMHA       840
                : ::*   *  ::*: ****:  *:::**  .:. .:             : :.::::*: :*

MmTTLL5         SEAELEEVLTFYTQKNKSASVFLGTHSKSSKNSSSYSDSGAKGDHPETIQEVKIKQPKQQ       868
DmTTLL5         QESDLEAVLTLHTNVTGIMPFLYNRCGLSV-PPTPPIPSGLHGF--------LRALPSMV       891
                .*::** ***::*: .     .:     . *     :    ** :*             *.

MmTTLL5         QATEIHA--DKLSRFTTSSGKEAKLVYTNCSSFCSPAAVLLQRLPSSHLSSVITTSALSA       926
DmTTLL5         SSTGINRELSKYDGYFKNFDKEKVFL----------------------------------       917
                .:* *.    .* . : ..   **  ::

MmTTLL5         GPGHHASLSQIPPAVPSLPHQPALLLSPVPDNAPPSIHSGTQNVSPAGLPRCRSGSYTIG       986
DmTTLL5         ------------------------------------------------------------       917

MmTTLL5         PFSSFQSAAHIYSQKLSRPSSAKAAGSCHPHKHHSGIAKTQKEGEDVSLNRRYNQSLVTA       1046
DmTTLL5         ------------------------------------------------------------       917

MmTTLL5         ELQRLAEKQAARQYSPASHISLLTQQVTNLNLASSVINRSSASTPPTLRPVISPSGPTWS       1106
DmTTLL5         ------------------------------------------------------------       917

MmTTLL5         IQPDLHASETHSSPPGSRSLQTGGFAWEGEVENNAYSKTTGVVPQHKYHPTAGSYQLHFA       1166
DmTTLL5         ------------------------------------------------------------       917

MmTTLL5         LQQLEQQKLQSRQLLDQSRARHQAIFGSQTLPNSSLWTMNNGPGCRISSATTGGQKPNTL       1226
DmTTLL5         ------------------------------------------------------------       917

MmTTLL5         PQKVVAPPNSSTLVSKPASNHKQVLRKPASQRASKGSSAEGQLNGLQSSLNPAAFMPITN       1286
DmTTLL5         ------------------------------------------------------------       917
MmTTLL5         STGSLEAPQVIFARSKPLPTQSGALATVIGQRKSKSVKSGTI     1328
DmTTLL5         ------------------------------------------     917
```

# Supplementary Figure 2 – Full blots from Figure 2a

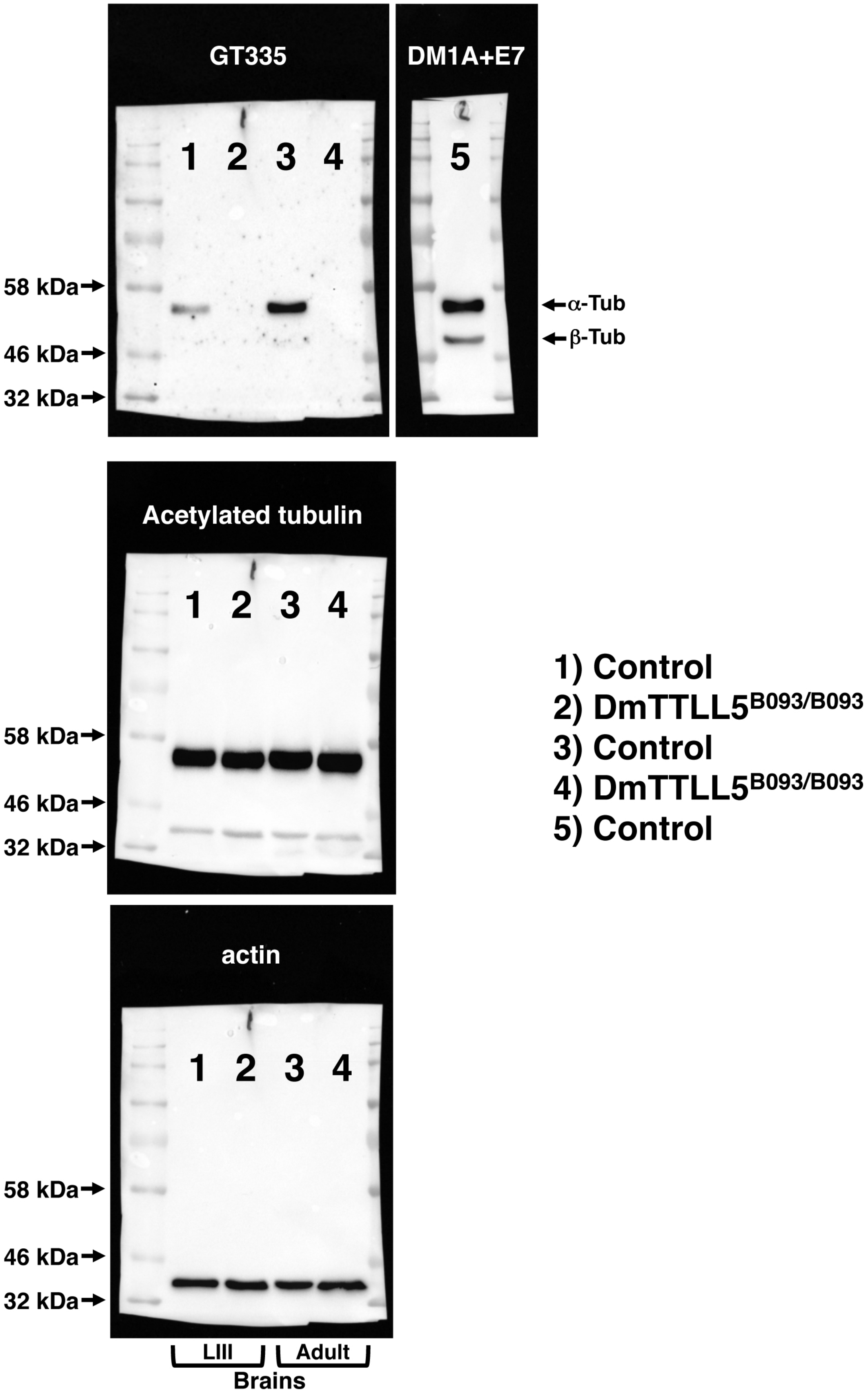

## Supplementary Figure 3 – Full blots from Figure 2b

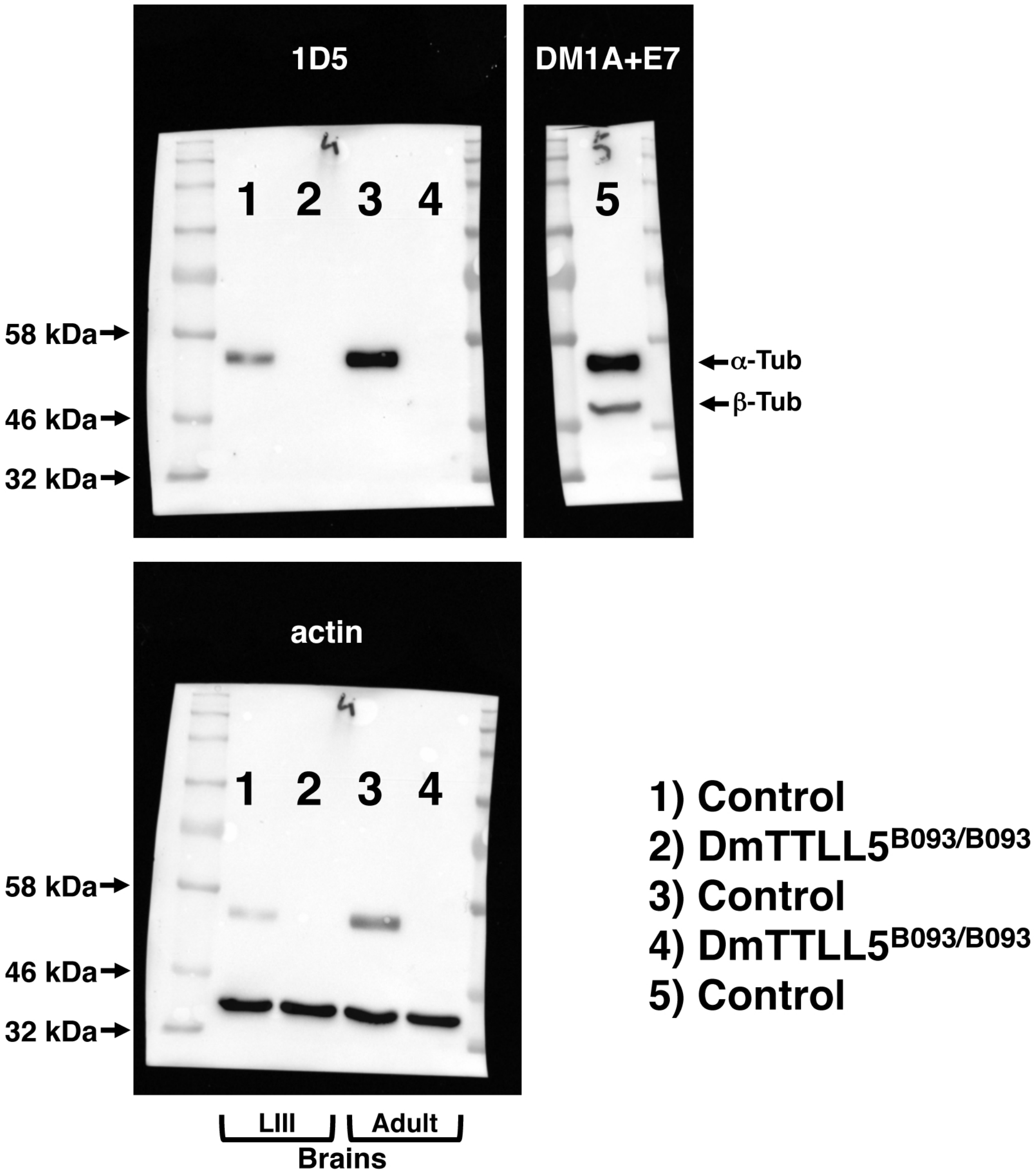

# Supplementary Figure 4 – Full blots from Figure 2c

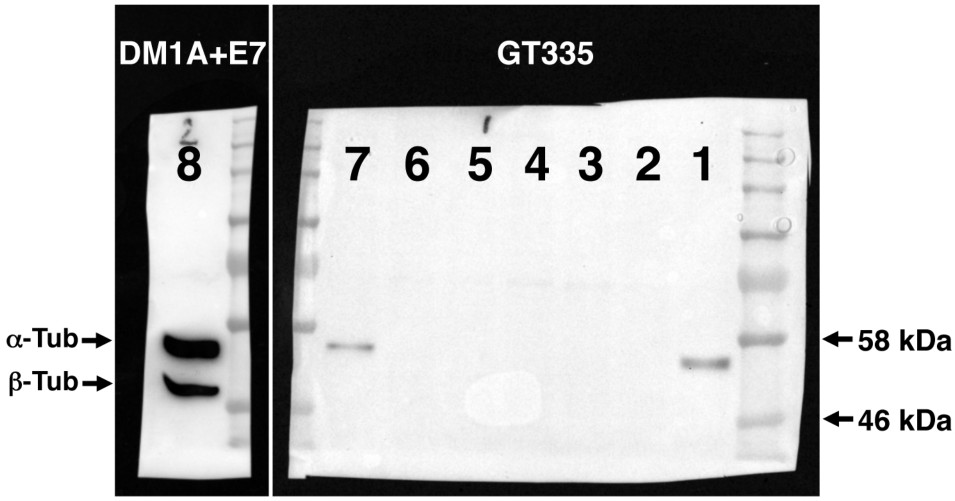


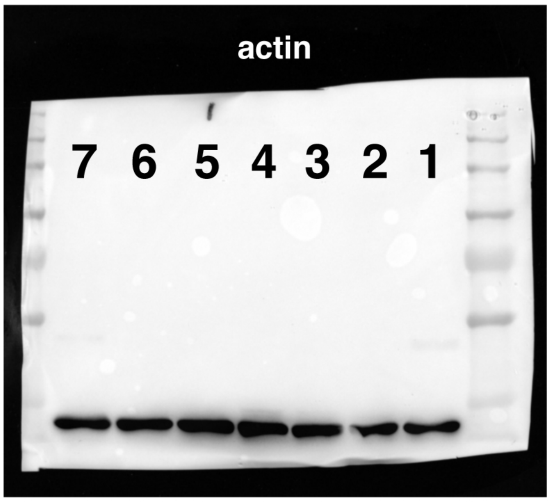


Adults Heads
1) Control
2) DmTTLL5$^{B093/B093}$
3) DmTTLL5$^{MI01917/MI01917}$
4) DmTTLL5$^{B093/MI01917}$
5) DmTTLL5$^{B093}$/Df(3R)BSC679
6) DmTTLL5$^{MI01917}$/Df(3R)BSC679
7) Rescue
8) Control

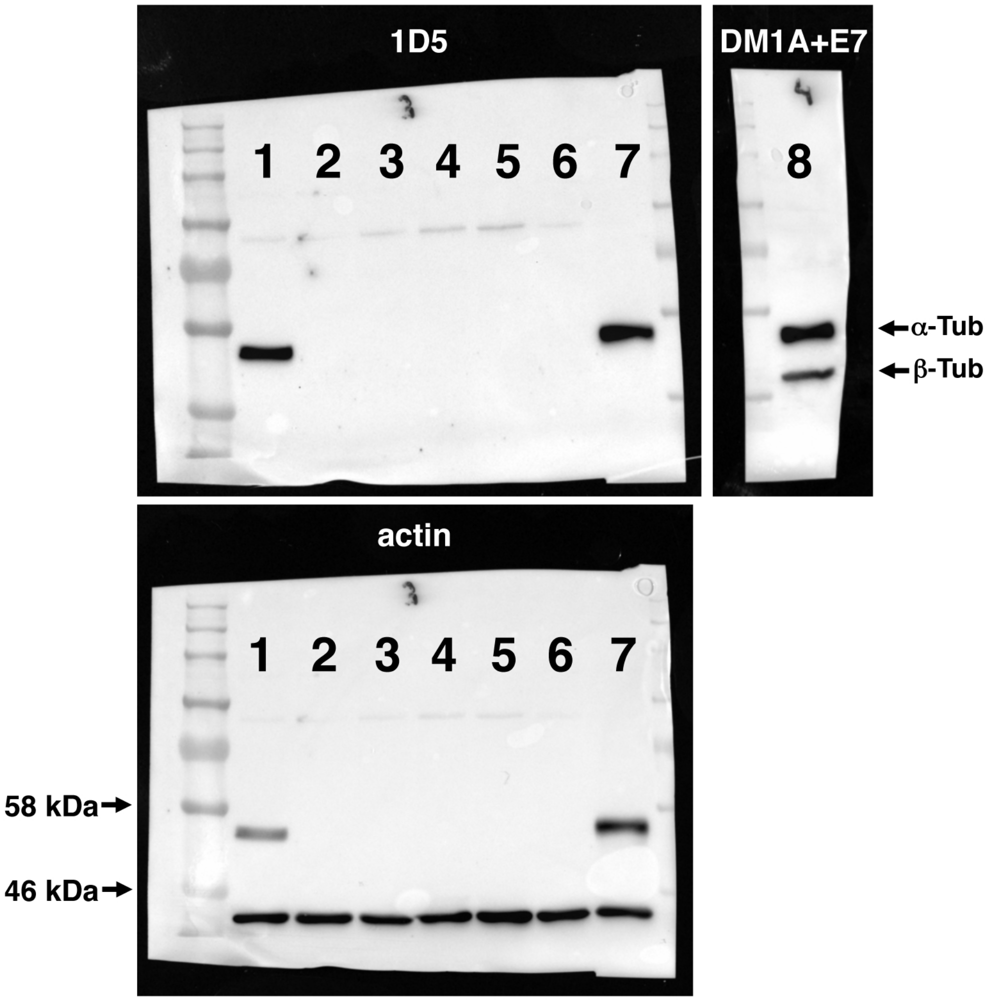

## SUPPLEMENTARY FIGURE LEGENDS

**Supplementary Figure 1: Mouse and *Drosophila* TTLL5 protein sequence alignment and annotations.**
Protein sequence of *Mus musculus* TTLL5 (MmTTLL5, accession number: NP_001074892) was obtained from the Ensembl database (www.ensembl.org) while the orthologous *Drosophila melanogaster* sequence (CG31108/DmTTLL5, accession number: NP_733081) was obtained from Flybase (http://flybase.org/). Sequences were aligned using Clustal Omega. Identical residues are underlined with asterisks (*). Strongly and weakly similar residues are underlined with double (:) and simple (.) dots, respectively. The extended TTL domain is outlined in green. The core TTL domain is underlined. The domains interacting with ATP and $Mg^{2+}$ are outlined in yellow with the region for ATP binding with essential Glutamate in bold (E366 in MmTTLL5). Residues of the cationic microtubule binding domain (c-MTBD) are in orange. The predicted substrate interacting domains are in blue. The receptor interaction domain of MmTTLL5, which is in purple, is absent in DmTTLL5.

**Supplementary Figure 2:** Full blots from Figure 2a.

**Supplementary Figure 3:** Full blots from Figure 2b.

**Supplementary Figure 4:** Full blots from Figure 2c.
